# Inflammation-Based Scores: A New Method for Patient-Targeted Strategies and Improved Perioperative Outcome in Cancer Patients

**DOI:** 10.1155/2014/142425

**Published:** 2014-04-27

**Authors:** Dario Bugada, Massimo Allegri, Patricia Lavand'homme, Marc De Kock, Guido Fanelli

**Affiliations:** ^1^Department of Anaesthesia and Intensive Care, Foundation IRCCS Policlinico San Matteo, P.le Golgi 19, 27100 Pavia, Italy; ^2^SIMPAR (Study in Multidisciplinary Pain Research) Group, Italy; ^3^Department of Surgical, Medical, Diagnostic and Pediatric Science, University of Pavia, Via Aselli 45, 27100 Pavia, Italy; ^4^Pain Therapy Service, Foundation IRCCS Policlinico San Matteo, P.le Golgi 19, 27100 Pavia, Italy; ^5^Department of Anesthesia and Perioperative Medicine, Catholic University of Louvain, St. Luc Hospital, 10 Avenue Hippocrate, 1200 Brussels, Belgium; ^6^Department of Anesthesia and Intensive Care, University of Parma, Via Gramsci 14, 43126 Parma, Italy

## Abstract

Systemic inflammatory response (SIR) has actually been shown as an important prognostic factor associated with lower postoperative survival in several types of cancer. Thus, the challenge for physicians is to find specific, low-cost, and highlyreliable inflammatory markers, clearly correlated with prognosis and able to preoperatively stratify patient's risk. Inflammation is a promising target to improve perioperative outcome, and data show that anti-inflammation techniques have a great potential in the perioperative period of cancer surgery. Inflammation scores could be useful to stratify patients with a potential better response to anti-inflammation strategies. Furthermore, inflammation scores could prevent failure of clinical trials by a better definition of patients to be included in such trials; inflammation scoring could clarify the real role of different drugs and techniques on outcome after cancer surgery, defining if different therapies are required for different patients. The role of this review is to focus on the currently available scores, in order to clarify their rationale and to analyze the actual evidence and limits, providing physicians with an updated overview of the possible inflammation-based prognostic scores for cancer patients undergoing surgery.

## 1. Introduction 

Inflammation plays a key role in cancer physiology, as it could promote carcinogenesis, dedifferentiation, and primary tumour growth [1]; furthermore, it promotes tumour cells proliferation by inhibiting apoptosis and increasing mitosis rate [1].

Inflammation has also some protective effects, participating in the initial anticancer response, mainly by cell-mediated immunity; immune cells can recognize factors produced by the inflammatory response in tumour to detect lymphocytes, macrophages, and dendritic cells (the so-called paradox of inflammation) [2]; inflammation has a causative role in many tumours and is a concomitant event in malignant recurrence [1].

As the role of inflammatory response is strictly connected with cancer physiology, many studies have investigated its role in cancer outcome. Regarding cancer surgery, systemic inflammatory response (SIR) has actually been shown as an important prognostic factor [3–57] associated with lower postoperative survival in several types of cancer.

Considering this prognostic value, the new challenges for physicians are represented by detecting degree of inflammation in each patient through specific inflammatory markers, clearly correlated with prognosis and able to preoperatively recognize immune changes for a better stratification of patient's risk. As inflammation is a complex phenomenon involving many cells and cytokines both systemically and locally (within the tumour), finding a low-cost, highly reliable, and easy to detect marker is often difficult. So far, a number of different prognostic scores based on inflammation have been proposed, with an increasing evidence of their value but with limits in their sensitivity and specificity. The role of this review is to focus on the currently studied and validated scores, in order to clarify their rationale and to analyze the actual evidence and limits, providing physicians with an updated overview of the possible inflammation-based prognostic scores for cancer patients undergoing surgery.

## 2. Methods

We systematically identified reports of studies assessing the use of inflammation-based scores. PubMed and MEDLINE databases were searched until November 2013 using the terms: “platelet to lymphocyte ratio” or “neutrophil to lymphocyte ratio” or “Glasgow prognostic score” and “cancer outcome.” Only papers in English were considered. Additional reports were identified from reference lists of retrieved papers. Only full papers were considered.

Included studies were both prospective and retrospective; retrospective analyses of prospective data were included and listed as retrospective (Tables 2 and 3).

Principal outcome measures were overall survival, disease-free survival, time to recurrence, pathology-free survival, relapse-free survival, mortality, and tumor resectability.

We included studies in which a selected marker was demonstrated to have a prognostic value alone or in combination with other markers (not strictly connected with inflammation).

We included studies involving only human subjects, both surgical and nonsurgical patients.

## 3. Results

The initial electronic literature searches revealed 136 studies, and, after evaluation of the abstracts, 78 were identified as potentially meeting the inclusion criteria. Evaluated inflammatory markers were NLR (neutrophil to lymphocyte ratio), PLR (platelet to lymphocyte ratio), GPS (Glasgow prognostic scale), and mGPS (modified GPS). 58 studies were excluded because they were about (1) inflammation markers other than NLR, PLR, and (m) GPS, (2) cancer noninflammation markers, (3) prognostic but not inflammation-based scores specifically relating to a single type of tumor, (4) cancer survival associated with different chemotherapies, and (5) effect of “other” variables on patient's outcome (infections, BMI, cigarette smoke, nutritional status, depression, psychological interventions, different surgical approaches, hemodynamic instability, reoperations, and graft histologic patterns). One study was excluded because it was not in English, while one study was excluded because it was a poster abstract of a scientific meeting. 36 additional studies were identified from reference lists of retrieved papers; additional evaluated markers were COP-NLR and thrombocytosis. Finally, 114 studies were considered for this review. Regarding (m) GPS, we retrieved more studies than we list in the references part of this paper; we decided to cite only some of them because they are the most recent, and their results summarize what was demonstrated by previous published papers.

### 3.1. Glasgow Prognostic Score

Glasgow prognostic score (GPS) measures acute-phase protein markers of the SIR, namely, C-reactive protein and albumin using standard thresholds (>10 mg/L for C-reactive protein and <35 g/L for albumin), which were combined to form a cumulative inflammation-based prognostic score [58]. This was subsequently refined to form the modified Glasgow prognostic score (mGPS) [59] when, in patients with primary operable colorectal cancer, hypoalbuminemia alone was found to have the same prognostic value as a GPS of 0 (Table 1). The higher the score is, the higher the risk is; an increased mGPS was predictive of a reduced cancer-specific survival in all cancers [56].

GPS is considered as a measure for systemic inflammation and reflects some of the biological changes, in both immune response and nutritional status, associated with cancer patients. The connection between systemic inflammation and mGPS is mainly acted through interleukin 6 (IL-6) role, as it is a pleiotropic cytokine with many physiological actions. In fact, IL-6 promotes not only CRP upregulation, but also albumin downregulation in the liver, as well as protein synthesis [60] and thrombocytosis [61]; it is also important to underline that some of these characteristics are related to nutritional status, because an elevated CRP level, hypoalbuminemia, and low BMI reflect cachexia due to hypercytokinemia resulting from tumour progression.

The evidence that the inclusion of a leukocyte count may add prognostic value to the validated mGPS and the recent introduction of high-sensitivity C-reactive protein measurements in routine clinical laboratory analysis (with threshold sensitivity lowered to 0.05 mg/L) has further modified the C-reactive protein/albumin combination improving the prognostic value derived from the components of a differential leukocyte count (neutrophil, lymphocyte, and platelet counts),  as demonstrated in a recent paper in which the addition of neutrophil and platelet counts, as well as a high-sensitivity C-reactive protein, enhanced the prognostic value of the mGPS [57].

GPS has been reported, in more than 60 studies (>30,000 patients), with independent prognostic value in patients with cancer in a heterogeneous variety of clinical scenarios and tumour types [62].

Currently, the GPS/mGPS is the most extensively validated one of the systemic inflammation-based prognostic scores and therefore may be used in the routine clinical assessment of patients with cancer, in addition or in preference to the current definitions of cachexia, together with tumour staging [62].

This biomarker not only identifies patients at risk for poorer prognosis but also provides a well-defined therapeutic target for treatment and future clinical trials. For example, patients with elevated mGPS scores should be considered in a precachexia status and offered with multimodal therapy (surgical tumour excision, anti-inflammation strategies, and metabolic and nutritional surveillance), which may delay the onset of cachexia and/or death [63]; anesthetic and surgical techniques able to reduce the enhanced inflammatory reaction after surgery could be planned in patients with higher basal inflammation. As a consequence, this will highlight the need to treat not only the tumour itself but also the SIR, a potentially more tractable target compared with well-established weight loss and/or poor performance status. Further studies are required to define the value of GPS/mGPS as a stratification factor, as selection criteria in randomized clinical trials, and as a therapeutic target in patients with cancer [62].

### 3.2. Neutrophil to Lymphocytes Ratio

Neutrophil to lymphocytes ratio (NLR) is one of the most studied inflammation prognostic markers for postoperative outcome. In fact, as it is well related to inflammation response, its role in identifying high-risk patients has been proposed in cancer and noncancer patients (see Table 2).

Different studies have highlighted the role of a high NLR as a preoperative tool to detect cancer patients with poorer prognosis, in terms of both general comorbidities and cancer disease-free and overall survival [64]. Furthermore, some studies have identified NLR as a valid tool to identify patients who are more sensitive to specific chemotherapy regimen both in the surgical and nonsurgical setting [5, 16, 28, 33, 35, 45, 55].

NLR is simple and reliable, being part of the standard exams for clinical evaluation, and can be assessed both preoperatively and postoperatively. Even if the majority of studies evaluate this marker in the preoperative setting, its prognostic value has been associated also with postoperative value, in terms of short-term morbidity and long-term mortality [20, 23, 28, 39, 43, 46, 49].

The relation between NLR and cancer outcome is probably to be found in tumour-associated immune changes; an elevated NLR reflects a decreased lymphocyte-mediated immunity (with an alteration in CD4+ helper/CD8+ suppressors ratio) and an increased production of inflammatory agents such as vascular endothelial growth factor (VEGF) that promotes tumour growth [65]; some studies evidence that the highest values of NLR are associated with aggressive tumour profiles [52]. Other studies focus on the NLR trend during tumour history, showing that a persistently high value is associated with poorer outcome (Table 2); all these findings suggest a probable direct connection between cancer biology and systemic inflammation expressed by NLR.

Even though most of the studies are retrospective, they reach the same conclusions. There are currently few prospective studies assessing the prognostic value of NLR, and a lack of homogeneity still exists in the definition of a standard NLR cutoff associated with different prognoses (Table 2); maybe different cutoffs have to be established for different types of cancer, as each type of cancer is associated with different changes in immune response.

### 3.3. The Role of Reactive Thrombocytosis: Platelet Count, COP-NLR, and Platelet to Lymphocyte Ratio

Recent studies have demonstrated that reactive thrombocytosis is associated with lower survival after surgery for several types of cancer [66–69], and platelet count could be related to the SIR, even if it is not still known which is the exact link between platelet, inflammation, and cancer outcomes. Reactive thrombocytosis is induced in a background of hypercytokinemia related to tumour progression. Among several inflammatory cytokines, IL-6 plays an important role in reactive thrombocytosis  [61]. IL-6 has a cell-proliferative effect, triggering the differentiation of megakaryocytes to platelets in the bone marrow [70, 71].

Although the normal platelet count is 15–30 × 10^4^ mm^3^, the cutoff value for reactive thrombocytosis is not clearly defined. However, most previous studies have used a cutoff value of 30–40 × 10^4^ mm^3^ [72, 73]. More studies are needed also to confirm the validity of this cutoff value.

Thrombocytosis is also induced from the tumour itself [74]. Generally, thrombocytosis is a laboratory finding in 10–57% of patients with malignancy, as a variety of neoplastic cells can stimulate platelet activation [75, 76]; some studies have revealed that cancer cells secrete vascular endothelial growth factor (VEGF), which also stimulates megakaryocyte differentiation [77]. Because VEGF induction promotes tumour growth [74, 78], thrombocytosis indirectly reflects tumour progression; a high level of VEGF is found in serum, platelets, and leukocytes of patients with malignant disease [79], and platelet interactions with malignant cells promote metastasis  [80].

Shimada et al. [69] have reported an association between high NLR and high platelet count in gastric cancer prognosis and also found that reactive thrombocytosis  [81] was associated with lower postoperative survival in patients with esophageal cancer. Other studies confirmed a high platelet count as a negative prognostic factor for renal cell, pancreatic, and colorectal cancers [72, 73, 82–84].

These results gave strong support to the use of a combination of reactive thrombocytosis and the NLR for prediction of postoperative survival; in a recent work by Ishizuka et al.  [85] presented the COP-NLR (combination of olatelet count and neutrophil to lymphocyte ratio), for predicting the postoperative survival of patients with colorectal cancer. In their study patients were divided in 3 groups based on presence or absence of platelet count >300.000/mm^3^ and NLR > 3 and a statistically significant difference in cancer specific survival were retrieved, with COP-NLR being able to classify patient's outcome into three independent groups. Moreover, COP-NLR resulted associated with tumour-related characteristics (type and dimension, invasivity and metastasis, operative curability, CEA) and SIR-related characteristics (high CRP, hypoalbuminemia, low BMI, and high NLR). These findings suggest the new COP-NLR as a new inflammation-based prognostic marker to complement classical tumour staging, reflecting biological changes related to high levels of IL-6 (the theoretically ideal marker of tumour-related SIR, whose serum levels are often difficult to be measured in the clinical practice). As there is only this retrospective study, more data are needed to confirm its predictive value demonstrating its specificity in other types of cancer. Nevertheless COP-NLR seems a valuable and promising tool for patient stratification in colorectal cancer.

Another prognostic marker related to platelet proliferation is the platelet to lymphocyte ratio (PLR).

PLR has been identified as a prognostic marker in patients with advanced gastric cancer [55] treated with chemotherapy (identifying more sensitive patients to specific type of chemotherapy), and its preoperative value correlated with prognosis (Table 3). Like NLR, studies are mainly retrospective and about only a few types of cancer; moreover, a lack of homogeneity still exists in the definition of a standard PLR cutoff associated with different prognoses. Further studies are needed to understand the real role of this marker and if it has a higher or lower prognostic value relating to NLR [6, 7].

## 4. Discussion 

As inflammation is strictly related to cancer, SIR is demonstrated to influence cancer patients' outcome.

In the last decade research focused its attention on finding specific biomarkers able to quantify systemic inflammation, in order to stratify patients' risk and detect patients more prone to be treated with different therapies, especially in cancer patients undergoing surgery.

These markers are not always simple or cheap to measure, so efforts have been done to find a low-cost, highly reliable, and easy to detect biomarker, clearly associated with prognosis and easily evaluated with routine laboratory analysis. Existing evidence suggests that a higher prognostic value is associated not with a single marker but with a combination of them; inflammation-based scores reflect many biological changes connected with cancer, and the impact of systemic inflammation on various aspects of patient's physiology [86, 87].

Among these inflammation-based scores, GPS is the most extensively validated one. It may be used in the routine clinical assessment of patients with cancer (particularly due to its strict connection with cachexia and poor performance status), suggesting that inflammation is a further therapeutic target, potentially able to delay the onset of cachexia and/or death in cancer patients.

Other scores reflect cancer-associated immune changes focusing on specific mechanisms connected with host-defense and tumour progression (NLR, thrombocytosis); regarding NLR, initial findings about a correlation with tumour aggressiveness were retrieved  [52], and a possible role not only of the preoperative value itself, but also of the whole NLR trend during patient history was observed [28, 39, 43, 46, 49], suggesting a possible role as a follow-up marker (to be demonstrated in proper designed trials).

Different evidence is available for each of them as prognostic scores, but limits still exist.

Firstly, most of the studies are retrospective, and prospective evaluations are needed to confirm the literature data. Moreover, a better definition of cutoff values has to be pursued; actual values are very heterogeneous and sometimes differ in the same study between different centers [23]; finally, it is still to be defined if different cancers are associated with different cutoffs or there is a common marker for prognosis to be used for all types of tumour. Investigators have just exceptionally compared effectiveness of different scores on predicting prognosis [6, 7]; however, further and more homogeneous studies are warranted to understand which score is more efficient in predicting the level of systemic inflammation and prognosis in each specific type of cancer, also according to tumour stage.

However, despite limits, there is a rising evidence of the validity of prognostic inflammation-based scores and biomarkers. Their analysis allowed stratifying the subgroup of patients and to understand cancer-related physiological mechanisms [87], configuring the first step towards a better multimodal therapy that, apart from cancer eradication, takes into account other targets to prolong patients' survival.

Inflammation is a promising target to improve perioperative outcome, and data show that anti-inflammatory techniques have a great potential in the perioperative period of cancer surgery [88]; evidence exists for a higher cytokine activation* in vitro* in patients with higher BMI (proinflammatory condition) [89]. The ability to quantify inflammation could allow identifying patients more at risk of enhanced response and hypercytokinemia in the postoperative period; inflammation scores could be useful to stratify patients more at risk or with a potential better response to anti-inflammatory strategies (and more worth to be treated with them), considering that the effect of NSAIDs and potentially all other anti-inflammatory techniques may depend on systemic inflammation level.

Regarding research, inflammatory scores could prevent failure of clinical trials testing drug candidate [90] and minimize unuseful exposure to ineffective therapies [90]. Inflammation scoring could clarify the real role of different drugs and techniques on outcome after cancer surgery, defining if different therapies are required for different patients. Forget et al. [23] showed that a single intraoperative administration of NSAIDs (ketorolac and diclofenac) could counteract effects of inflammation on cancer outcome and that this effect is twice greater in patients with NLR > 4 than in the whole series. Conversely, tumours with a slow growth, typically with a low level of inflammation and a small risk of early relapse, could have a smaller benefit of NSAIDs. This observation was done in a large retrospective series of 1,111 prostate cancer patients [91] and highlights the possible ability for NLR to guide drug choice on a specific target of patients. If data will be confirmed (NCT01806259) we could have indication to use NSAIDs on subtypes of patients in order to improve oncological outcome.

Moreover, discrepancies between studies focusing on the effects of regional analgesia or morphine in the perioperative period exist [92]; this could be explained by their different efficacy in patients with different systemic inflammation. Inclusion of inflammation analysis in future clinical trials could clarify which patients are more indicated to be treated with regional techniques.

Finally, as opioids were advocated to have immunodeppressive action, able to facilitate tumour dissemination [92], it would be useful to identify patients with enhanced inflammatory response, in order to understand if opioid therapy has beneficial effects on outcome in this specific subgroup of patients.

## Figures and Tables

**Table 1:** Glasgow Prognostic score and modified Glasgow Prognostic Score.

	Points
GPS	
CRP ≤ 10 mg/L and albumin ≥ 35 g/L	0
CRP > 10 mg/L	1
Albumin < 35 g/L	1
CRP > 10 mg/L and albumin < 35 g/L	2
MODIFIED GPS	
CRP ≤ 10 mg/L and albumin ≥ 35 g/L	0
CRP > 10 mg/L	1
CRP > 10 mg/L and albumin < 35 g/L	2

CRP: C-Reactive Protein.

**Table 2:** Published studies about neutrophil-to-lymphocyte ratio (NLR).

Author	Cancer type	Study nature	Cutoff	Outcome measure
Azab et al. [4]	Breast	Retrospective	3.3	NLR > 3.3 is predictor of higher mortality
Lee et al. [5]	gastric	Prospective	3	NLR normalization after one cycle of chemotherapy correlates with OS and PFS
He et al. [6]	Colorectal (metastatic)	Retrospective	3	NLR < 3 associated with better OS
Feng et al. [7]	Esophageal	Prospective	3.5	RFS, OS are not correlated with NLR
Stotz et al. [8]	Pancreatic	Retrospective	5	NLR > 5 associated with CSS
Absenger et al. [9]	Colorectal (stage II-III)	Retrospective	4	NLR > 4 associated to lower TTR
Gomez et al. [10]	HCC	Retrospective	5	Preoperative NLR > 5 was an adverse predictor of DFS/OS
Cho et al. [11]	Ovarian	Retrospective	2.6	NLR > 2.6 associated with lower OS and DFS
Kao et al. [12]	Mesothelioma	Retrospective	5	NLR > 5 associated with lower OS
Jung et al. [13]	Gastric	Retrospective	3	NLR > 3 predict worse OS/DFS
Kim et al. [14]	Thyroid	Retrospective	—	NLR is a negative prognostic factor in papillary thyroid carcinomas
Walsh et al. [15]	Colorectal	Retrospective	5	NLR greater than 5 correlated with OS/CSS
Hung et al. [16]	Colorectal	Retrospective	5	NLR > 5 associated with significantly worse OS and DFS
Halazun et al. [17]	Liver metastasis of colorectal cancer	Retrospective	5	NLR > 5 predictive for risk of death and recurrence
Tomita et al. [18]	NSCLC	Retrospective	2.5	NLR > 2.5 associated to lower survival at 5 years
Keizman et al. [19]	Renal	Retrospective	3	NLR < 3 associated with better PFS and OS
Kim et al. [20]	Uterine Sarcoma	Retrospective	2.12	NLR not correlated with PFS and OS, but good marker of progression
Sharaiha et al. [21]	Esophageal	Retrospective	5	NLR > 5 was associated with significantly worse DFS and OS
Garcea et al. [22]	Pancreatic	Prospective	5	NLR > 5 associated to lower DFS
Forget et al. [23]	Breast, Renal, Lung	Retrospective	4/3 3 5	High NLR, associated with poorer prognosis (mortality, recurrence)
Ong et al. [24]	pancreatic	Retrospective	—	Significantly higher in patients undergoing bypass at exploration for potentially curative carcinoma
Aliustaoglu et al. [25]	pancreatic	Retrospective	5	NLR > 5 associated with poor survival
Aliustaoglu et al. [26]	Gastric	Retrospective	2.56	NLR < 2.56 associated with higher survival
An et al. [27]	Pancreatic	Retrospective	5	NLR > 5 associated with shortened survival
Kishi et al. [28]	Liver metastasis of colorectal cancer	Retrospective	5	NLR > 5 correlated with OS. When chemotherapy normalizes high NLR, improved survival is expected.
Guthrie et al. [29]	Colorectal	Retrospective	5	NLR > 5 associated with lower OS
Fox et al. [30]	Renal	Retrospective	—	High NLR associated with OS
Mano et al. [31]	HCC	Retrospective	2.81	High NLR associated with poorer OS and DFS
Demirtaş et al. [32]	Bladder	Retrospective	2.5	NLR were not found to be independent predictor of prognosis
di Giacomo et al. [33]	Melanoma	Retrospective	—	NLR is a marker of response to chemotherapy
Szkandera et al. [34]	Soft tissue sarcoma	Retrospective	3.45/3.58	NLR > 3.45 associated with lower TTR NLR > 3.58 associated with lower OS
Kobayashi et al. [35]	Renal	Prospective	—	NLR predictor of response after targeted therapy
Dimitrascu et al. [36]	Cholangiocarcinoma	Retrospective	3.3	NLR > 3.3 associated with lower PFS
Yao et al. [37]	NSCLC	Retrospective	2.63	NLR > 2.63 associated with lower PFS and OS
Keizman et al. [38]	Prostate	Retrospective	3	NLR > 3 associated to lower PFS
McNally et al. [39]	HCC—TACE	Retrospective	—	Trend towards elevated NLR correlates with survival
Jeong et al. [40]	Gastric	Retrospective	3	NLR > 3 associated with poorer OS
Chua et al. [41]	Epithelial appendicular malignancy	Retrospective	2.6	NLR > 2.6 associated with lower PFS and OS
Carrhuters et al. [42]	Rectal cancer	Retrospective	5	NLR > 5 associate with lower OS, DFS, TTLR
Pinato et al. [43]	HCC—TACE	Retrospective	5	Persistently high NLR associate with worse survival
Chiang et al. [44]	Colorectal	Retrospective	3	NLR > 3 correlates with lower DFS
Sato et al. [45]	Esophageal	Retrospective	2.2	NLR > 2.2 correlates with higher recurrence
Chen et al. [46]	HCC—RFTA	Retrospective	2.4	Baseline high NLR predictor of poor OS—post procedural high NLR associated with poorer OS/higher risk of recurrence
Thavaramara et al. [47]	Ovarian	Retrospective	—	Higher NLR associated with poorer PFS
Huang et al. [48]	HCC—chemoembolization	Retrospective	3.3	NLR > 3.3 predicts poor survival
Chua et al. [49]	Colon	Retrospective	5	NLR > 5 correlated with lower OS; NLR normalization associated with higher PFS
Rashid et al. [50]	Esophageal	Retrospective	3.5	No correlations
Halazun et al. [51]	Liver transplantation for HCC	Retrospective	5	Elevated NLR significantly increases the risk for tumor recurrence and recipient death
Gomez et al. [52]	Intrahepatic cholangicarcinoma	Retrospective	5	NLR > 5 correlated with reduced DFS and aggressive tumor profile
Shibutani et al. [53]	Colorectal	Retrospective	2.5	NLR > 2.5 associated with poorer OS
Malik et al. [54]	Colorectal after resection of hepatic metastasis	Retrospective	5	NLR > 5 is a negative prognostic factor
Lee et al. [55]	Lung	Retrospective	—	A high post treatment NLR is associated with poor prognosis. An early reduction in the NLR after effective treatment may indicate survival improvement in patients with poor prognosis.

OS: overall survival; DFS: disease free survival; PFS: progression-free survival; CSS: cancer-specific survival; TTR: time to recurrence; TTLR: time to local recurrence.

**Table 3:** Published studies about platelet-to-lymphocyte ratio (PLR).

Author	Cancer type	Study nature	Cutoff	Outcome
Bhatti et al. [93]	Pancreatic	Retrospective	<100 100–200 >200	No correlation between PLR and OS
Smith et al. [94]	Pancreatic	Retrospective	<150 151–300 >300	Higher PLR correlates with lower OS
Sakka et al. [95]	Periampullary	Retrospective	300	PLR > 300 associated with decreased survival
Smith et al. [96]	Periampullary	Retrospective	160	Higher PLR combined with CA19.9 predicts decreased survival
Smith et al. [97]	Pancreatic	Retrospective	150	PLR useful predictor for tumor resectability (combined with CA 19.9)
Lee et al. [55]	Gastric	Prospective	160	PLR normalization after one cycle of chemotherapy correlates with OS and PFS
He et al. [6]	Colorectal	Retrospective	<150 151–300 >300	Higher PLR correlates with worse PFS and OS. NLR better prognostic factor than PLR
Feng et al. [7]	Esophageal	Prospective	150	PLR > 150 associated with decreased RFS and OS
Chua et al. [41]	Appendicular epithelial malignancy	Retrospective	166	PLR > 166 associated with lower OS and PFS
Carrhuters et al. [42]	Rectal	Retrospective	160	PLR > 160 associated with lower OS, DFS and TTLR
Aliostaouglu et al. [26]	Gastric	Retrospective	160	PLR < 160 associated with significantly higher survival

OS: overall survival; DFS: disease free survival; PFS: progression-free survival; RFS: relapse-free survival; TTLR: time to local recurrence.
